# Gene Flooding: Proposal to Flood Invasive Populations With Inbred Individuals as a Form of Low‐Tech Genetic Control

**DOI:** 10.1002/ece3.72913

**Published:** 2026-01-19

**Authors:** John Gould, Chad Beranek

**Affiliations:** ^1^ School of Environmental and Life Sciences University of Newcastle Callaghan New South Wales Australia

**Keywords:** bottleneck, *Gambusia*, genetic drift, inbreeding depression, invasive species

## Abstract

Genetic controls are at the cutting edge of invasive species management whereby modified individuals are released into target populations to induce declines by disrupting their reproductive potential. Yet, such methods are not always feasible without considerable costs and expertise. We propose an alternative, low‐tech genetic approach that reduces the genetic diversity of invasive wild populations by flooding them with related individuals from an inbred colony that have been derived from a single ancestral line. We refer to this process as ‘gene flooding’ and explore its potential use to control invasive mosquitofish, *Gambusia* spp. Using this hypothetical approach, the repeated release of inbred individuals across multiple generations inflicts sustained genetic bottlenecking on a target population as the frequency of gene variants from the wild population are diluted in the gene pool, causing saturation with a small subset of gene variants derived from the inbred colony. Our simulation of gene flooding demonstrates evidence of its capacity to cause the loss of wild type genetics and to keep a wild population in a suspended genetic state thereafter, because it is being pumped with a static allele pool and continuously over many generations. These processes suppress the population's ability to adapt to evolutionary pressures it experiences in the habitat it has invaded. It may be possible to disrupt the genetic integrity of small and isolated invasive populations using low‐tech genetic controls such as gene flooding, which requires real‐world testing.

## Graphical abstract

Genetic controls are at the cutting edge of invasive species management but are not always feasible without considerable costs and expertise. We propose an alternative, low‐tech genetic approach that reduces the genetic diversity of wild populations by flooding them with related individuals from an inbred colony that have been derived from a single ancestral line. We refer to this process as ‘gene flooding’ and explore its potential use to control invasive mosquitofish, *Gambusia* spp. using genetic simulations, which show it could have the capacity to disrupt the genetic integrity of targeted populations.

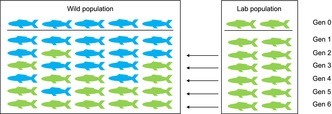

## 1 Introduction

The establishment of non‐native species outside their natural range is a key threatening process that has contributed to the decline of native species worldwide (Gurevitch and Padilla 2004). Once introduced, an invader may pose a threat to native species via exposure to novel predation and competition pressures, disease and ecosystem disturbance (Berger et al. 1999; Doherty et al. 2016; Eldridge et al. 2019). While some native species can coexist with invaders or have adapted in response to this new selecting pressure (e.g., Parrott et al. 2019), others remain vulnerable and require ongoing assistance to prevent continued declines (Dickman 1992; Legge et al. 2018).

Physical methods have been established to manage invasive species (e.g., Denny and Dickman 2010), as well as their effect on native species (e.g., Legge et al. 2018), which are based on the direct killing or movement of individuals from a defined area. Physical methods to control invasive populations are not feasible for large populations with extensive ranges, restricting most successful eradications to islands (Spatz et al. 2022). Biological methods involve the deliberate introduction of non‐native species to act as controls and are more feasible for large populations than physical controls as they may only require a single release into the system, but their effect may inadvertently spill over onto native species (Messing and Wright 2006). As managers continue to struggle with the spread of invasive species, novel approaches are needed that offer supplementary or more effective lines of defence (Gould et al. 2024).

Genetic controls are a pioneering method for the management of invasive species and currently involve the release of specific individuals into wild populations to disrupt their reproductive potential (Harvey‐Samuel et al. 2017). For example, gene drive techniques involve the release of genetically manipulated individuals that breed in wild populations to spread deleterious genes, leading to population decline (Wedell et al. 2019). Unlike biological controls, modified individuals may need to be released repeatedly into target populations dependent on the technique that is utilised (Harvey‐Samuel et al. 2017). These methods are also logistically unfeasible for widespread use without considerable funding, resources and expertise, and carry ethical and permitting issues related to the release of modified individuals.

An alternate genetic‐based approach may be to proactively reduce the genetic diversity of invasive populations by flooding them with related individuals derived from a single ancestral line. We refer to this process as ‘gene flooding’, which we hypothesise could induce inbreeding depression, genetic drift and maladaptation by mimicking the effects of a declining population. This is conceptually an action that is the direct opposite to the conservation management of threatened populations, whereby release programmes are undertaken to preserve/boost genetic diversity (Tracy et al. 2011). We explore this hypothetical management strategy of gene flooding, the theory behind how it could work genetically, and how it could be used in conjunction with other control measures using a case study of an invasive fish.

## 2 The Genetics of Populations

### 2.1 The Genetic Risks of Small Populations

Population declines and/or isolation caused by human or natural disturbances, as well as foundation events, can lead to bottlenecking events whereby genetic diversity is lowered due to small population sizes (Frankham et al. 2002). This decline in genetic diversity can occur due to genetic drift, whereby gene variants (alleles), even beneficial ones, may be lost by chance as individuals are removed from the population via mortality or during reproduction if they happen not to be inherited. This drift can cause alleles at individual loci to become fixed and occurs more often in smaller populations due to the greater influence of chance sampling effects (e.g., fewer pairings mean fewer chances for each allele type to be inherited). Additionally, breeding between closely related individuals is more likely in smaller populations and increases the chance of offspring inheriting two copies of ‘hidden’ recessive deleterious alleles that become fixed, leading to their expression and resulting in reduced fitness via inbreeding depression (Charlesworth and Willis 2009; Keller and Waller 2002). Small populations can thus suffer a spiralling effect (extinction vortex) where they become less resilient to changing environments as they lack genetic diversity to be adaptable, while the accumulation of deleterious alleles decreases individual fitness or causes lethal abnormalities in offspring (Blomqvist et al. 2010; Charlesworth and Willis 2009; DeRose and Roff 1999), further reducing population size.

Populations trapped in an extinction vortex can be revived through the reintroduction of extinct/novel alleles, either by immigration (e.g., release of captive individuals via translocations and assisted colonisation) or beneficial mutations over longer periods. Yet, it is possible for small, inbred populations to persist and escape an extinction vortex (Weiser et al. 2016), particularly if deleterious alleles have not yet drifted to fixation and are purged from the gene pool via natural selection (Robinson et al. 2018). Large populations can also have low genetic diversity, such as after a bottlenecking event, and persist despite generally lower fitness (e.g., O'Brien et al. 1986). It is the quality of alleles still extant and the length of time populations remain small that influence the extinction risk posed by the genetic consequences of declines, by influencing the risk of exposure to genetic drift and inbreeding depression.

### 2.2 The Genetics of Invasive Populations

Invading populations often show a lack of genetic diversity (polymorphic loci and heterozygosity) relative to source populations via founding effects that should reduce adaptive potential (Dlugosch and Parker 2008). However, this is not apparent in all invading populations (Wares et al. 2005), being dependent on the genetic diversity and number of founders. Additionally, whilst invading populations are often small, leading to an increased risk of genetic drift, this risk declines as populations increase in size (Nei et al. 1975). Even if there is a loss of genetic variation due to the founding effect, invading populations may still show strong adaptive potential if selection leads to changes in in the frequency of alleles that remain and favours those that provide an advantage. This is especially true if the population is supplied with additional alleles with further migration events from source or other invading populations that counteract founding effects and allow for admixture of divergent lineages and novel gene combinations (Dlugosch and Parker 2008; Estoup et al. 2016). The drivers of genetic diversity in invading populations are complex and this must be considered when applying genetic‐based control measures.

## 3 Gene Flooding to Control Invasive Species

### 3.1 Proposed Methodology

The process of gene flooding would begin by extracting a single male/female pair (generation 0) from a target wild population to establish a laboratory colony. The offspring of this pair (generation 1) would be kept together to force reproduction to occur between siblings. This would result in the production of a first generation of inbred offspring (generation 2) that are only able to interbed amongst themselves. This process would be continued over subsequent generations to create an increasingly large and inbred colony from a single ancestral line. With each generation, a subset of individuals (e.g., 20%) would be extracted and released back into the wild population. The repeated process of inbreeding and release would flood the wild population with related individuals over consecutive generations, causing the wild population to become increasingly like the laboratory population in terms of its genetic composition.

### 3.2 Hypothesised Impacts of Gene Flooding

**FIGURE 1:**
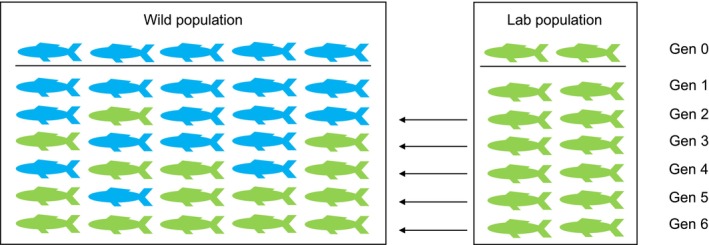
Stimulating genetic diversity loss by flooding a wild population with individuals from an inbred laboratory population. Conceptual diagram shows a laboratory population of individuals (green) derived from a single mating pair, and a wild population of individuals (blue). In each generation, a percentage of inbred individuals from the lab are released into the wild population, causing the flushing of wild‐type alleles from the wild population.

We hypothesise that the process of gene flooding could have three critical effects on a target population:

*Genetic flushing*—It may force a population through a genetic bottleneck in the absence of a population size bottleneck, compromising its reproductive potential without needing to directly manage animal numbers (Figure 1). This would occur as the repeated release of laboratory individuals alters the proportion of alleles in the wild population over time. In particular, the frequency of alleles originally derived from the wild population are diluted in the gene pool as it becomes saturated with the small subset of alleles derived from the laboratory colony. This would reduce the genetic diversity of the wild population by causing the gene pool to become dominated by a small number of alleles and increasing the chance of wild type alleles being lost by chance in a process akin to genetic drift more commonly observed in small populations (Hufbauer and Roderick 2005), despite the target population remaining large.

**FIGURE 2:**
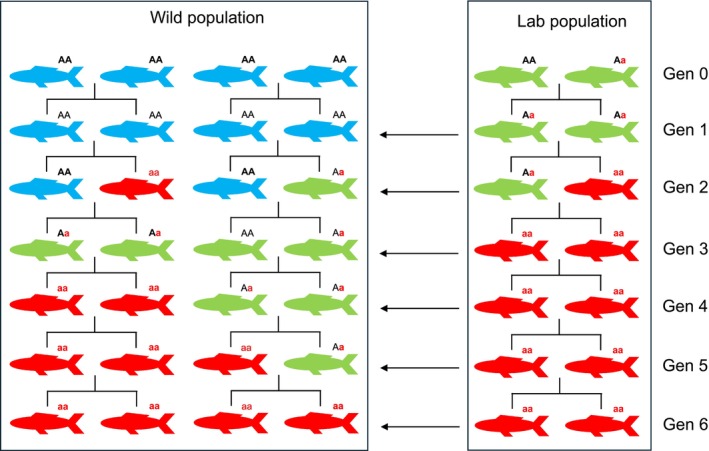
Stimulating inbreeding depression in a wild population via release of individuals from an inbred laboratory population. Conceptual diagram shows a laboratory population of individuals (green) derived from a single mating pair, with one individual possessing a deleterious allele (a) and a wild population of individuals (blue) lacking the deleterious allele. Individuals that have inherited two copies of the ‘hidden’ recessive and deleterious allele are shown in red. In each generation, a percentage of the inbred individuals from the laboratory are released into the wild population, causing the spread of the deleterious allele as the wild population becomes increasingly inbred.

*Inbreeding depression*—It may increase the spread and rate of fixation of deleterious alleles due to inbreeding depression (Figure 2). In the laboratory population, inbreeding over many generations can cause more offspring to carry deleterious alleles. These deleterious alleles are transferred to the wild population with each laboratory release, where interbreeding increases their frequency over time. This could reduce the survival and reproductive capacity of individuals that are relatively less fit, causing the population to decline. However, a potential limitation to this effect would be the purging of the deleterious allele via natural selection, which would counter this effect by reducing genetic load.

*Induced maladaptation*—It may inhibit the ability of a population to adapt to environmental conditions if it prevents the selection and increased frequency of alleles that give rise to favourable traits. This occurs as the flooding with alleles derived from the laboratory population filters out wild type alleles while also keeping the wild population in a static genetic state. This disrupts the natural selection process by preventing beneficial alleles from becoming common over generations. This could induce maladaptation akin to outbreeding depression (Hufbauer and Roderick 2005), as the repeat releases inflates the occurrence of the laboratory type alleles in the wild population.

## 4 Case Study: Controlling *Gambusia*

The eastern mosquito fish, *Gambusia holbrooki*, and the closely related *G. affinis* (referred to as *Gambusia* hereon in) have been deliberately introduced to several countries to control mosquitoes (White and Pyke 2011). It has since naturally dispersed into connected waterways and is now considered one of the most invasive animals in the world (Lowe et al. 2000). This fish impacts naïve systems as both a veracious predator of and superior competitor to native species (White and Pyke 2011). It is difficult to manage once present due to its biological attributes; it is relatively small, can disperse in shallow water (< 3 mm; Alemadi and Jenkins 2008), is highly fecund (clutch sizes range from 1 to over 300), gives birth to live young and has rapid maturation (1–2 months from being born to reproductively mature), which allow for rapid population increase (Leberg 1993; Pyke 2005; White and Pyke 2011). The fish can also tolerate a range of conditions, such as water salinity, and has a high tolerance for environmental stress (Hubbs 2000), affording it an ability to exist in multiple different aquatic ecosystems (Gould et al. 2024).

Current methods of *Gambusia* eradication include the drying of waterbodies (O'Meara and Darcovich 2008) and use of poisons (Thompson and Thompson 2022; White and Pyke 2008). Drying is the most widely used method but can be difficult to achieve as *Gambusia* can survive in low levels of water, requiring waterbodies to remain dry for several weeks (Pollard et al. 2017). The artificial and repetitive draining of wetlands can have negative effects on these systems, such as causing reductions in species richness of native freshwater macroinvertebrate communities (Hanford et al. 2020).

### 4.1 Control Using Gene Flooding

Many of the biological attributes of *Gambusia* that have made it a successful coloniser are also what make it a potential candidate for control using gene flooding. Its small size, lack of sensitivity to fluctuating conditions, generalist diet, rapid maturation and high fecundity would allow for (i) a laboratory population to be rapidly set‐up with limited space and time, with low demands in terms of resource provisioning, and (ii) a wild population to be flooded with a high number of inbred individuals over consecutive generations that overwhelms the gene pool in a short period of time. It has also been shown that the genetic diversity of introduced *Gambusia* populations are lower than for native populations (Grapputo et al. 2006), suggesting they may be relatively easy to gene flood. *Gambusia* are also found in aquatic systems that can be hydrologically isolated, such as freshwater ponds (Gould et al. 2024), which are ideal for applying gene flooding as a form of control by minimising chances of genetic rescue from migration.

## 5 Simulating Gene Flooding

### 5.1 Simulation Set‐Up

We simulated the effects of gene flooding on the genetics of two hypothetical *Gambusia* populations (wild and laboratory). To do so, we used Monte Carlo methods of repeated random sampling in a forward‐time simulation in R version 4.3.1 (R Team 2023), to model stochastic evolutionary processes that influence population genetics, such as sexual selection, genetic drift, inheritance, survival and culling at a single locus. We acknowledge that our modelling does not incorporate polygenic traits, epistatic interactions or environmental variability, which influence real‐world population genetics. This simulation has been conducted to illustrate our theoretical control method and reveal general patterns of its effect on target populations.

Within both the wild and laboratory populations, individuals were paired randomly and only once per reproductive round, with unpaired individuals unable to reproduce in cases where there was an uneven population size. To account for the large clutch size of *Gambusia* (White and Pyke 2011), each pairing gave rise to 10 offspring, with each offspring inheriting a single allele from each parent. Inherited alleles were sampled randomly from the parental genotypes for each offspring to reflect the probabilistic nature of Mendelian inheritance. To account for the capacity of *Gambusia* adults to reproduce several times (up to nine broods per year; White and Pyke 2011), we allowed individuals to survive and participate in breeding for up to three consecutive generation before dying, thus giving rise to overlapping generations. In each generation, individual survival was random except for the effect of fitness differences. The carrying capacity of both the wild and laboratory populations was set to 1000. Although this represents relatively small populations, it is within the confines of real‐world populations of *Gambusia* in small water bodies; density ranges from 1 to 60 individuals/m^2^ (Zulian et al. 1995), leading to population sizes of 50 to 3000 individuals in a 50 m^2^ pond. When populations exceeded their carrying capacity, individuals were randomly culled to maintain numbers below or at this threshold size. This pruning did not account for the potential effects of natural selection, such as differences in survival probability between individuals based on their respective fitness. The simulation was run for 100 generations and repeated 10 times.

*Laboratory population*: It was initially set at two individuals to represent a single mating pair originating an inbred line, with the genotypes L1‐L2 and L1‐L2 across all simulation runs. L2 was selected as a deleterious allele that had negative fitness consequences on sexual selection, reproductive output and survival. To simulate the effects of sexual selection, individuals homozygous for the deleterious allele had a reduced chance of reproducing (80%). After sexual selection, matings were random with respect to an individual's genotype. To simulate the effects of inbreeding avoidance, individuals that were related as parent–offspring or full sibs had a 30% chance of pairing, while half sibs had a 50% chance of pairing. To simulate the potential effects of inbreeding depression, the L2 allele was made deleterious to survival and reproduction when individuals inherited two copies (homozygous L2‐L2) and thus expressed its inferior qualities. An individual carrying two copies of L2 had a 50% reduction in survival each generation relative to all other individuals. These individuals also had reduced reproductive output that was dependent on the genotype of their partner. If a single parent was homozygous for L2, the total number of offspring produced was reduced by 30%. If both parents were homozygous for L2, the output was reduced by 80%.

*Wild population*: It was initially set at 100 founding members. The alleles for the founders were unique to those of the laboratory population (W1, W2), with the genotypes of founders randomly selected from this allele pool and randomly for each simulation run. In each generation, 20% of the laboratory population was randomly selected and released into the wild population, akin to one‐way migration. This process simulated the introduction of the laboratory‐based alleles into the wild population with gene flooding. Once released, the laboratory‐derived individuals were given the same breeding restrictions as wild individuals.

### 5.2 Simulation Findings

**FIGURE 3:**
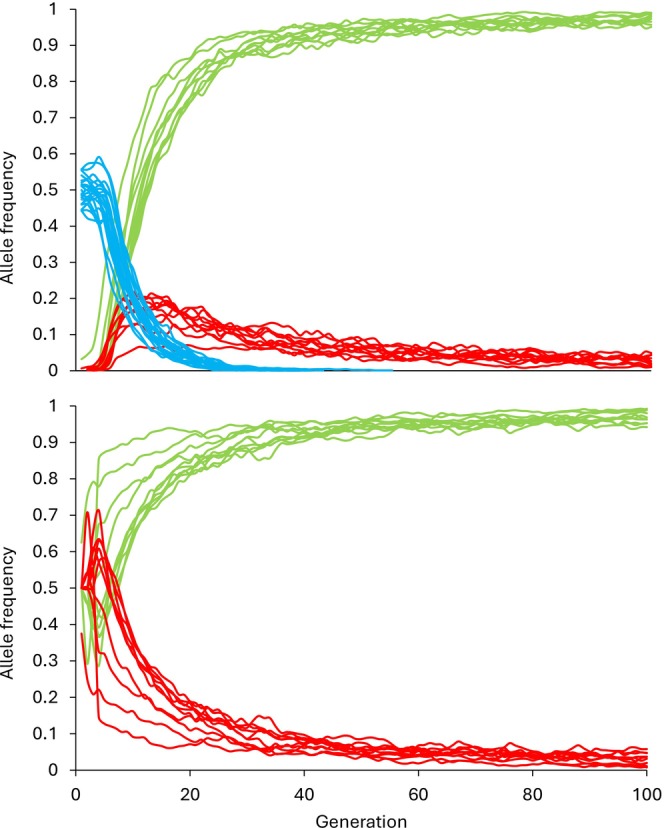
Effect of gene flooding on simulated *Gambusia* populations. Temporal changes in the frequency of occurrence of wild‐type alleles W1 and W2 (both represented by blue lines), and laboratory type alleles L1 (green line) and L2 (red line). Laboratory alleles were introduced into the wild population (part a) via the repeat release of inbred individuals from the laboratory population (part b), with the allele L2 being deleterious on individual fitness. Allele frequencies were tracked for 100 generations.

The wild type alleles W1 and W2 were rapidly lost from the wild population, with the first wild allele in each run lost after a mean of 34 generations (SD = 5) and the second allele lost after 39 generations (SD = 4); accounting for *Gambusia* gestation (1–2 weeks) and maturation (1–2 months) (Pyke 2005), this would equate to time periods of 8.5 and 9.75 years, respectively. Both the laboratory type alleles L1 and L2 showed an initial increase in frequency over time. Allele L1 became the most common allele after an average of 11.5 generations (SD = 2.0), equating to 2.9 years, and continued to increase in frequency to 0.97 (SD = 0.01) after 100 generations. The occurrence of the deleterious allele L2 peaked at 0.18 (SD = 0.05) at generation 13 (SD = 4), before a gradual decline to 0.03 (SD = 0.01) after 100 generations (Figure 3A).

In the laboratory population, the non‐deleterious allele L1 rapidly increased in frequency across all runs, from 0.5 (SD = 0) to 0.97 (SD = 0.02) after 100 generations. In contrast, the deleterious allele L2 rapidly decreased in frequency across all runs, from 0.5 (SD = 0) to 0.03 (SD = 0.02) but was not entirely lost from any run after 100 generations (Figure 3B).

## 6 Outcomes of Gene Flooding

### 6.1 Impacts on Target Populations

Our simulation results provide support for our hypothesis that gene flooding could lead to genetic diversity loss in wild populations, both in terms of allele presence and dominance. This is because the repeat introduction of laboratory alleles across generations overwhelms the wild gene pool, inflicting sustained genetic bottlenecking by flushes wild alleles from the population and saturating it with a small subset of alleles derived from the inbred laboratory population. Additionally, our simulation also provides support for our other hypothesis that gene flooding maintains target population in a static genetic state after the loss of wild type alleles, given that it is being pumped with a small allele pool derived from the laboratory population and iteratively over consecutive generations. These processes could make target populations easier to control or possibly eradicate if the loss of genetic diversity and inability for fitter alleles to be selected makes them more susceptible to changing environmental conditions, stochastic events, or novel pressures such as climate change (Jump et al. 2009), as it leads to the loss of beneficial alleles that disrupts the natural selection process and induces maladaptation (Blomqvist et al. 2010; Charlesworth and Willis 2009; DeRose and Roff 1999; Hufbauer and Roderick 2005). However, it appears that gene flooding is unlikely to induce population collapse due to inbreeding depression as deleterious alleles derived from the laboratory population do not accumulate and become fixed but rather purged from the wild population due to natural selection. Nevertheless, our findings suggest that genetic drift can be induced in populations via gene flooding, which could allow for the extinction of wild type alleles from invading populations without the need for reducing population size itself. We would expect target populations would be exposed to gene flooding until the loss of wild type alleles or even heterozygosity. This is a form of low‐tech genetic regulation that could be used to knock out favourable genes from wild populations, with the alleles targeted differing between populations to make each maladaptive to their respective environmental conditions.

There are several population parameters that dictate population size and whether inbreeding leads to the accumulation of deleterious alleles and a subsequent crash; (1) larger clutch sizes reduce the negative consequences of having any offspring within a given clutch that happen to inherit two copies of a deleterious allele, as there is likely to be other offspring within that same clutch that do not inherent two copies of that deleterious allele if the parents are heterozygotes, (2) rapid increases and maintenance of a population's size will reduce the effect of genetic drift and maintain allele frequencies between generations, (3) the capacity for individuals to reproduce several times and inter‐generational pairings (e.g., fathers with daughters) provide for larger effective population sizes, slowing the fixation of deleterious alleles by backcrossing younger, more inbred individuals with their older, less inbred relatives that still possess gene variants that have been lost from more recent generations, reviving alleles that are potentially lost in newer generations and (4) the relatively lower survival and reproductive output of individuals expressing deleterious allele leads to their purging from the population via natural selection, further reducing the risk of them becoming fixed. While this finding suggests a limitation of the effect of gene flooding on wild populations, it is a positive finding as it provides clear evidence that a viable laboratory population could be sustained even from a single inbred ancestral line without suffering the compounding effects of inbreeding depression. However, this is only possible if the laboratory population increases in size rapidly and remains sufficiently large, which have a higher chance of being facilitated by large clutch sizes in species such as *Gambusia*, optimal conditions to prevent mortality and inter‐generational breeding. This is important, as the laboratory colony will suffer the consequences of inbreeding depression at a faster pace than wild populations being targeted.

### 6.2 Issues to Resolve

It is possible that inbred individuals released into the wild population are (i) less fit and thus less likely to reproduce or produce viable offspring, or (ii) unfavoured as partners by individuals in the wild population. This has been observed in *Gambusia*, where inbred male sperm is less likely to fertilise female gametes and are outcompeted by outbred male sperm in instances of multiple paternity (Marsh et al. 2017; Vega‐Trejo et al. 2017). There is also evidence for reduced female fecundity when mating with related males (Vega‐Trejo et al. 2015). These conditions could give rise to complications if they reduce the rate of inbreeding in wild populations being targeted while outbred individuals still remain. However, our simulation findings suggest that, even if a portion of released individuals suffering from inbreeding depression have lower reproductive potential and survival, by chance their siblings may not if they inherit different allele combinations (e.g., still heterozygous).

Additionally, it is possible that individuals may avoid mating with closely related individuals (Scribner and Avise 1993), which could impede the growth of the laboratory colony if individuals are less inclined to reproduce. The effect on the wild population could be threefold; (1) related individuals that are released are less inclined to partner amongst themselves, instead selecting non‐related individuals from the wild population, causing the rapid mixing of the two gene pools and the rapid spread of the laboratory gene variants that would cause offspring in subsequent generations to become related, (2) once the laboratory gene pool is spread across the entire wild population it may result in fewer matings that causes a more rapid population decline or (3) it may cause the least related individuals within the population to be favoured for reproduction, helping to preserve gene variants from the wild population and reducing the rate of inbreeding depression. While this may reduce the applicability of this method to many species, it has been shown that *Gambusia* females do not avoid breeding with related males (Vega‐Trejo et al. 2015) and inbreeding does not affect male traits associated with female mate choice (Kahn et al. 2010; Vega‐Trejo, Head, and Jennions 2016; Vega‐Trejo, Jennions, and Head 2016). Our simulation also suggests that there is no decline in the wild population due to gene flooding, which goes against point two, and that wild type alleles are not preserved in the population but rather purged quite rapidly, which is evidence against point three.

Other biological attributes will need to be considered and overcome for gene flooding to be applied and this may restrict its application to specific species and environmental contexts. For example, *Gambusia* females can store the sperm of multiple partners and carry broods that have been multiply sired (Robbins et al. 1987; Zane et al. 1999). *Gambusia* can produce large broods (see Pyke 2005), and it has been suggested that a single female can start a new population with no damaging founder effects if she has stored sperm from several males (Alemadi and Jenkins 2008). This capacity for large broods, sperm storage and multiple paternity could have several implications on the use of gene flooding; (1) any female individuals collected from the wild population as founders of the laboratory colony would need to be virgins, and (2) in the wild population, multiple paternity may allow for the retention of higher genetic variation over time that would slow the rate of wild‐type alleles being driven to extinction. The former issue is resolved via the selection of virgins while the latter could be reduced by implementing gene flushing when populations have already been reduced, such as during periods of drought, thus reducing the potential number of male–female pairing combinations possible.

Population level effects of inbreeding have been reported in *Gambusia* by Leberg (1990), who found smaller population sizes with smaller males when founded by siblings compared to unrelated pairs. However, this contrasts with Kandl (2001) who found no effect of inbreeding on population size in experimental populations founded by 10 siblings (Kandl 2001). Kandl (2001) suggests that inbreeding effects may have been masked by family effects, whereby some lineages may happen to have higher survival or fecundity than others if they possess relatively more advantageous alleles (Kandl 2001). This could suggest that the founding mating pairs selected for the laboratory colony should be tested for genetic diversity and fitness, while the first generation of the laboratory colony should be a sibling pair derived from parents and possibly also grandparents that are also siblings, to establish a population from a stock with both low allelic diversity and heterozygosity. There is also a risk of selecting relatively fitter individuals from the wild population to start the laboratory population and thus spreading those genes upon release into the wild population. Yet, the genetics of the laboratory population would not be novel, as these individuals would be derived from the wild population they are being released back to. However, it would be valuable to identify inferior genotypes prior to selection to further reduce the adaptability of the wild population being gene flooded.

This method requires flooding an already invaded ecosystem with even more invasive individuals, risking greater ecological damage during the release phase and potential emigration of the invasive individuals. Other genetic control methods have less potential for unwanted population growth because offspring are nonviable or skew the population sex ratio (Harvey‐Samuel et al. 2017). Nonetheless, there may be situations where managers would be interested in this method as a means of controlling invasive species that are already isolated and difficult to remove via other methods, or already present in large numbers or at carrying capacity where the risk of additional environmental damage would be reduced. Additionally, the captive breeding requirements for the inbred colony may exceed the costs to implement other control methods. There are also animal ethics considerations as gene flooding purposely results in individuals bearing genotypes that could make existence suboptimal. This may still be better than poisoning which can cause painful deaths and have ecosystem wide impacts on non‐target species (Twigg and Parker 2010).

### 6.3 Feasibility and Criteria

**FIGURE 4:**
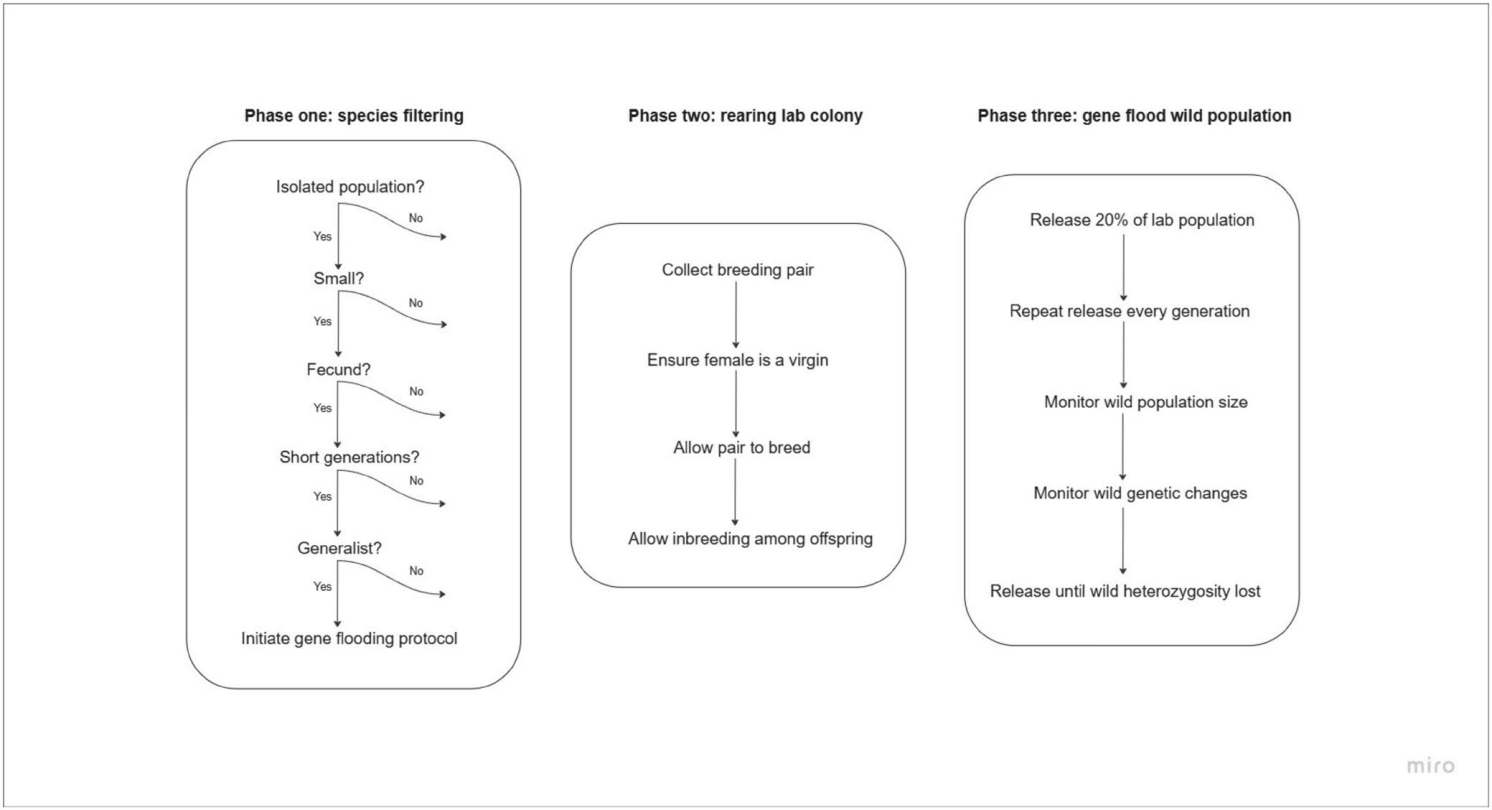
Assessing target pest species as candidates for gene flooding. Workflow diagram showcasing how a manager may select species for gene flooding based on their attributes and environmental context.

Gene flooding would require the sustained release of inbred individuals over several generations to impact allele frequencies significantly. Thus, the effectiveness of gene flushing will be influenced by the biological attributes of the invading species, as well as the attributes of target populations and their environmental contexts. Species with large dispersing capabilities or connected populations increase the chance of emigration that would reverse the effects of gene flooding via gene flow and the continuous introduction of alleles that have been lost. It is thus unlikely that this method would be feasible for species that have the capacity for long distance dispersal or exploit highly connected systems, such as aerial species. However, we believe it would be effective against species that occur in isolated populations, such as those confined to ponds or islands, given the physical isolation that prevents genetic recovery. Target species should be highly fecund with a short generation time, in order to sustain a large laboratory population and for the rapid release of large numbers of individuals into wild populations over many generations in a feasible time frame to reduce resource investment needs. This is shown for *Gambusia*, where the time for wild populations to become overwhelmed by laboratory‐based alleles was as little as 2.5 years. The method would thus be limited to species with short generation times. Finally, the method would likely be most effective for relatively small populations, such as those in small water bodies. We have provided a workflow diagram, showcasing how a manager may assess whether a target pest species is a good candidate for gene flooding based on species attributes and environmental context (Figure 4).

Sustaining a large laboratory population of inbred individuals that have a high genetic load may be biologically and logistically problematic. As deleterious alleles accumulate, individuals are more likely to suffer reduced fitness. Yet, our simulation shows that the large size of the laboratory population limits the effect of inbreeding depression, much like it does for the wild population, with deleterious alleles not accumulating but rather being purged from the population. This would effectively allow the laboratory population to be sustained and remain viable over many generations for application.

## 7 Conclusion

We have proposed the idea of gene flooding not as a panacea but as a tool which could be implemented on a case‐by‐case basis to help mitigate the impacts of some invasive species. Extensive testing is required to determine the true efficacy of this method, particularly related to species life history, population attributes and environmental conditions, as well as further genetic simulations that can provide insight into the inter‐generational impacts on the genetic diversity of invasive populations. This hypothetical technique needs to be trialled to ultimately determine if its effects on can be sufficient to contribute to the control of invasive populations.

## Author Contributions

**John Gould:** conceptualization (equal), data curation (lead), formal analysis (lead), investigation (lead), methodology (lead), visualization (lead), writing – original draft (equal), writing – review and editing (equal). **Chad Beranek:** formal analysis (supporting), methodology (supporting), writing – original draft (equal), writing – review and editing (equal).

## Conflicts of Interest

The authors declare no conflicts of interest.

## Supporting information


**Data S1:** ece372913‐sup‐0001‐supinfo.docx.

**Data S2:** ece372913‐sup‐0002‐supinfo.xlsx.

## Data Availability

Data have been provided as Supporting Information.

## References

[ece372913-bib-0001] Alemadi, S. D. , and D. G. Jenkins . 2008. “Behavioral Constraints for the Spread of the Eastern Mosquitofish, *Gambusia holbrooki* (Poeciliidae).” Biological Invasions 10: 59–66.

[ece372913-bib-0002] Berger, L. , R. Speare , and A. Hyatt . 1999. “Chytrid Fungi and Amphibian Declines: Overview, Implications and Future Directions.”

[ece372913-bib-0003] Blomqvist, D. , A. Pauliny , M. Larsson , and L. Å. Flodin . 2010. “Trapped in the Extinction Vortex? Strong Genetic Effects in a Declining Vertebrate Population.” BMC Evolutionary Biology 10: 1–9. 20122269 10.1186/1471-2148-10-33 PMC2824661

[ece372913-bib-0004] Charlesworth, D. , and J. H. Willis . 2009. “The Genetics of Inbreeding Depression.” Nature Reviews Genetics 10, no. 11: 783–796. 10.1038/nrg2664 19834483

[ece372913-bib-0005] Denny, E. A. , and C. Dickman . 2010. Review of Cat Ecology and Management Strategies in Australia. Invasive Animals Cooperative Research Centre.

[ece372913-bib-0006] DeRose, M. A. , and D. A. Roff . 1999. “A Comparison of Inbreeding Depression in Life‐History and Morphological Traits in Animals.” Evolution 53, no. 4: 1288–1292. 28565531 10.1111/j.1558-5646.1999.tb04541.x

[ece372913-bib-0007] Dickman, C. R. 1992. “Conservation of Mammals in the Australasian Region: The Importance of Islands.” In Australia and the Global Environmental Crisis: Looking for Peaceful Solutions, edited by J. N. Coles and J. M. Drew , 175–214. Academic Press.

[ece372913-bib-0008] Dlugosch, K. M. , and I. M. Parker . 2008. “Founding Events in Species Invasions: Genetic Variation, Adaptive Evolution, and the Role of Multiple Introductions.” Molecular Ecology 17, no. 1: 431–449. 17908213 10.1111/j.1365-294X.2007.03538.x

[ece372913-bib-0009] Doherty, T. S. , A. S. Glen , D. G. Nimmo , E. G. Ritchie , and C. R. Dickman . 2016. “Invasive Predators and Global Biodiversity Loss.” Proceedings of the National Academy of Sciences 113: 11261–11265. 10.1073/pnas.1602480113 PMC5056110 27638204

[ece372913-bib-0010] Eldridge, D. J. , S. K. Travers , J. Val , A. Zaja , and K. E. Veblen . 2019. “Horse Activity is Associated With Degraded Subalpine Grassland Structure and Reduced Habitat for a Threatened Rodent.” Rangeland Ecology & Management 72: 467–473.

[ece372913-bib-0011] Estoup, A. , V. Ravigné , R. Hufbauer , R. Vitalis , M. Gautier , and B. Facon . 2016. “Is There a Genetic Paradox of Biological Invasion?” Annual Review of Ecology, Evolution, and Systematics 47, no. 1: 51–72.

[ece372913-bib-0012] Frankham, R. , J. Ballou , and D. Briscoe . 2002. Introduction to Conservation Genetics. Cambridge University Press.

[ece372913-bib-0013] Gould, J. , A. Callen , C. Beranek , and C. McHenry . 2024. “The Only Way is Down: Placing Amphibian Ponds on Plateaux Protects Against *Gambusia* Colonization.” Restoration Ecology 32: e14159.

[ece372913-bib-0014] Grapputo, A. , A. Bisazza , and A. Pilastro . 2006. “Invasion Success Despite Reduction of Genetic Diversity in the European Populations of Eastern Mosquitofish (*Gambusia holbrooki*).” Italian Journal of Zoology 73, no. 1: 67–73.

[ece372913-bib-0015] Gurevitch, J. , and D. K. Padilla . 2004. “Are Invasive Species a Major Cause of Extinctions?” Trends in Ecology & Evolution 19, no. 9: 470–474. 16701309 10.1016/j.tree.2004.07.005

[ece372913-bib-0016] Hanford, J. K. , C. E. Webb , and D. F. Hochuli . 2020. “Management of Urban Wetlands for Conservation Can Reduce Aquatic Biodiversity and Increase Mosquito Risk.” Journal of Applied Ecology 57, no. 4: 794–805.

[ece372913-bib-0017] Harvey‐Samuel, T. , T. Ant , and L. Alphey . 2017. “Towards the Genetic Control of Invasive Species.” Biological Invasions 19: 1683–1703. 28620268 10.1007/s10530-017-1384-6 PMC5446844

[ece372913-bib-0018] Hubbs, C. 2000. “Survival of *Gambusia affinis* in a Hostile Environment.” Southwestern Naturalist 45, no. 4: 521–522.

[ece372913-bib-0019] Hufbauer, R. A. , and G. K. Roderick . 2005. “Microevolution in Biological Control: Mechanisms, Patterns, and Processes.” Biological Control 35, no. 3: 227–239.

[ece372913-bib-0020] Jump, A. S. , R. Marchant , and J. Peñuelas . 2009. “Environmental Change and the Option Value of Genetic Diversity.” Trends in Plant Science 14, no. 1: 51–58. 19042147 10.1016/j.tplants.2008.10.002

[ece372913-bib-0021] Kahn, A. T. , B. Mautz , and M. D. Jennions . 2010. “Females Prefer to Associate With Males With Longer Intromittent Organs in Mosquitofish.” Biology Letters 6, no. 1: 55–58. 19755529 10.1098/rsbl.2009.0637 PMC2817265

[ece372913-bib-0022] Kandl, K. L. 2001. “Effects of Inbreeding and Salinity Stress on Population Dynamics of Eastern Mosquitofish.” Transactions of the American Fisheries Society 130, no. 6: 1224–1232.

[ece372913-bib-0023] Keller, L. F. , and D. M. Waller . 2002. “Inbreeding Effects in Wild Populations.” Trends in Ecology & Evolution 17, no. 5: 230–241.

[ece372913-bib-0024] Leberg, P. 1990. “Influence of Genetic Variability on Population Growth: Implications for Conservation.” Journal of Fish Biology 37: 193–195.

[ece372913-bib-0025] Leberg, P. L. 1993. “Strategies for Population Reintroduction: Effects of Genetic Variability on Population Growth and Size.” Conservation Biology 7, no. 1: 194–199.

[ece372913-bib-0026] Legge, S. , J. C. Woinarski , A. A. Burbidge , et al. 2018. “Havens for Threatened Australian Mammals: The Contributions of Fenced Areas and Offshore Islands to the Protection of Mammal Species Susceptible to Introduced Predators.” Wildlife Research 45: 627–644.

[ece372913-bib-0027] Lowe, S. , M. Browne , S. Boudjelas , and M. De Poorter . 2000. 100 of the World's Worst Invasive Alien Species: A Selection From the Global Invasive Species Database (Vol. 12). Invasive Species Specialist Group Auckland.

[ece372913-bib-0028] Marsh, J. N. , R. Vega‐Trejo , M. D. Jennions , and M. L. Head . 2017. “Why Does Inbreeding Reduce Male Paternity? Effects on Sexually Selected Traits.” Evolution 71, no. 11: 2728–2737. 28857148 10.1111/evo.13339

[ece372913-bib-0029] Messing, R. H. , and M. G. Wright . 2006. “Biological Control of Invasive Species: Solution or Pollution?” Frontiers in Ecology and the Environment 4: 132–140.

[ece372913-bib-0030] Nei, M. , T. Maruyama , and R. Chakraborty . 1975. “The Bottleneck Effect and Genetic Variability in Populations.” Evolution 29: 1–10. 28563291 10.1111/j.1558-5646.1975.tb00807.x

[ece372913-bib-0031] O'Brien, S. J. , D. E. Wildt , and M. Bush . 1986. “The Cheetah in Genetic Peril.” Scientific American 254, no. 5: 84–95.3704622

[ece372913-bib-0032] O'Meara, J. , and K. Darcovich . 2008. “Gambusia Control Through the Manipulation of Water Levels in Narawang Wetland, Sydney Olympic Park 2003‐2005.” Australian Zoologist 34, no. 3: 285–290.

[ece372913-bib-0033] Parrott, M. L. , J. S. Doody , C. McHenry , and S. Clulow . 2019. “Eat Your Heart Out: Choice and Handling of Novel Toxic Prey by Predatory Water Rats.” Australian Mammalogy 42, no. 2: 235–239.

[ece372913-bib-0034] Pollard, C. J. , M. P. Stockwell , D. S. Bower , et al. 2017. “Removal of an Exotic Fish Influences Amphibian Breeding Site Selection.” Journal of Wildlife Management 81: 720–727.

[ece372913-bib-0035] Pyke, G. H. 2005. “A Review of the Biology of *Gambusia affinis* and *G. holbrooki* .” Reviews in Fish Biology and Fisheries 15: 339–365.

[ece372913-bib-0036] R Team . 2023. R: A Language and Environment for Statistical Computing. R Foundation for Statistical Computing.

[ece372913-bib-0037] Robbins, L. W. , G. D. Hartman , and M. H. Smith . 1987. “Dispersal, Reproductive Strategies, and the Maintenance of Genetic Variability in Mosquitofish (*Gambusia affinis*).” Copeia 1987: 156–164.

[ece372913-bib-0038] Robinson, J. A. , C. Brown , B. Y. Kim , K. E. Lohmueller , and R. K. Wayne . 2018. “Purging of Strongly Deleterious Mutations Explains Long‐Term Persistence and Absence of Inbreeding Depression in Island Foxes.” Current Biology 28, no. 21: 3487–3494. 30415705 10.1016/j.cub.2018.08.066 PMC6462144

[ece372913-bib-0039] Scribner, K. T. , and J. C. Avise . 1993. “Cytonuclear Genetic Architecture in Mosquitofish Populations and the Possible Roles of Introgressive Hybridization.” Molecular Ecology 2, no. 3: 139–149.

[ece372913-bib-0040] Spatz, D. R. , N. D. Holmes , D. J. Will , et al. 2022. “The Global Contribution of Invasive Vertebrate Eradication as a Key Island Restoration Tool.” Scientific Reports 12, no. 1: 13391. 35948555 10.1038/s41598-022-14982-5 PMC9365850

[ece372913-bib-0041] Thompson, G. G. , and S. A. Thompson . 2022. “Lessons Learned From the Use of Rotenone to Eradicate Feral Fish in Two Irrigation Lakes in Western Australia.” Ecological Management & Restoration 23, no. 2: 158–165.

[ece372913-bib-0042] Tracy, L. N. , G. P. Wallis , M. G. Efford , and I. G. Jamieson . 2011. “Preserving Genetic Diversity in Threatened Species Reintroductions: How Many Individuals Should be Released?” Animal Conservation 14: 439–446.

[ece372913-bib-0043] Twigg, L. , and R. Parker . 2010. “Is Sodium Fluoroacetate (1080) a Humane Poison? The Influence of Mode of Action, Physiological Effects, and Target Specificity.” Animal Welfare 19, no. 3: 249–263.

[ece372913-bib-0044] Vega‐Trejo, R. , M. L. Head , and M. D. Jennions . 2015. “Evidence for Inbreeding Depression in a Species With Limited Opportunity for Maternal Effects.” Ecology and Evolution 5, no. 7: 1398–1404. 25897379 10.1002/ece3.1445 PMC4395169

[ece372913-bib-0045] Vega‐Trejo, R. , M. L. Head , and M. D. Jennions . 2016. “Inbreeding Depression Does Not Increase After Exposure to a Stressful Environment: A Test Using Compensatory Growth.” BMC Evolutionary Biology 16: 1–12. 27036748 10.1186/s12862-016-0640-1 PMC4818490

[ece372913-bib-0046] Vega‐Trejo, R. , M. L. Head , J. S. Keogh , and M. D. Jennions . 2017. “Experimental Evidence for Sexual Selection Against Inbred Males.” Journal of Animal Ecology 86, no. 2: 394–404. 27973712 10.1111/1365-2656.12615

[ece372913-bib-0047] Vega‐Trejo, R. , M. Jennions , and M. Head . 2016. “Are Sexually Selected Traits Affected by a Poor Environment Early in Life?” BMC Evolutionary Biology 16: 1–12. 27905874 10.1186/s12862-016-0838-2 PMC5134236

[ece372913-bib-0048] Wares, J. P. , A. R. Hughes , and R. K. Grosberg . 2005. “Mechanisms That Drive Evolutionary Changes.” In Species Invasions: Insights Into Ecology, Evolution, and Biogeography, edited by D. F. Sax , J. J. Stachowicz , and S. D. Gaines , 229–257. Sinauer.

[ece372913-bib-0049] Wedell, N. , T. Price , and A. Lindholm . 2019. “Gene Drive: Progress and Prospects.” Proceedings of the Royal Society B 286, no. 1917: 20192709. 31847764 10.1098/rspb.2019.2709 PMC6939923

[ece372913-bib-0050] Weiser, E. L. , C. E. Grueber , E. S. Kennedy , and I. G. Jamieson . 2016. “Unexpected Positive and Negative Effects of Continuing Inbreeding in One of the World's Most Inbred Wild Animals.” Evolution 70, no. 1: 154–166. 26683565 10.1111/evo.12840

[ece372913-bib-0051] White, A. , and G. Pyke . 2008. “Frogs on the Hop: Translocations of Green and Golden Bell Frogs *Litoria aurea* in Greater Sydney.” Australian Zoologist 34, no. 3: 249–260.

[ece372913-bib-0052] White, A. , and G. Pyke . 2011. “World War II and the Rise of the Plague Minnow *Gambusia holbrooki* (Girard, 1859) in Australia.” Australian Zoologist 35: 1024–1032.

[ece372913-bib-0053] Zane, L. , W. S. Nelson , A. G. Jones , and J. C. Avise . 1999. “Microsatellite Assessment of Multiple Paternity in Natural Populations of a Live‐Bearing Fish, *Gambusia holbrooki* .” Journal of Evolutionary Biology 12, no. 1: 61–69.

[ece372913-bib-0054] Zulian, E. , A. Bisazza , and G. Marin . 1995. “Variations in Male Body Size in Natural Populations of *Gambusia holbrooki* .” Ethology Ecology & Evolution 7: 1–10.

