# Supplementary material for: Gene Flooding: Proposal to Flood Invasive Populations With Inbred Individuals as a Form of Low‐Tech Genetic Control
Source: Ecol Evol. 2026 Jan 19;16(1):e72913. doi: 10.1002/ece3.72913 (PMC12815697; doi:10.1002/ece3.72913)
Supplement: Supplementary file 1 — Data S1: ece372913‐sup‐0001‐supinfo.docx. [file ECE3-16-e72913-s002.docx]

rm(list=ls())

###############################################

# PARAMETERS

###############################################

generations <- 100

offspring_per_pair_lab <- 10

offspring_per_pair_wild <- 10

lab_capacity <- 1000

wild_capacity <- 1000

lab_alleles_pair1 <- c("A", "B")

lab_alleles_pair2 <- c("A", "B")

initial_alleles_wild <- c("D", "C")

###############################################

# SEXUAL SELECTION AGAINST BB GENOTYPES

###############################################

apply_sexual_selection <- function(pop) {

bb_mask <- pop$allele1 == "B" & pop$allele2 == "B"

keep_bb <- runif(sum(bb_mask)) < 0.8 # 80% mating chance

selected <- pop[!bb_mask, ]

if (sum(bb_mask) > 0) {

selected <- rbind(selected, pop[bb_mask, ][keep_bb, ])

}

return(selected)

}

###############################################

# PEDIGREE-BASED RELATEDNESS

###############################################

calculate_relatedness <- function(ind1, ind2) {

# Parent–offspring

if (ind1$ID %in% c(ind2$parent1, ind2$parent2) ||

ind2$ID %in% c(ind1$parent1, ind1$parent2)) {

return(0.5)

}

# Full siblings (share BOTH parents)

if (!is.na(ind1$parent1) && !is.na(ind2$parent1)) {

if (ind1$parent1 == ind2$parent1 &&

ind1$parent2 == ind2$parent2) {

return(0.5)

}

}

# Half siblings (share ONE parent)

if (ind1$parent1 %in% c(ind2$parent1, ind2$parent2) ||

ind1$parent2 %in% c(ind2$parent1, ind2$parent2)) {

return(0.25)

}

return(0) # Unrelated

}

###############################################

# Convert relatedness to mating probability

###############################################

inbreeding_mating_probability <- function(r) {

if (r >= 0.5) return(0.3) # parent–offspring, full sibs not forbidden but reduced to 30%

if (r >= 0.25) return(0.5) # half siblings: 50% chance

return(1) # unrelated: free mating

}

###############################################

# GENOTYPE EFFECTS ON FECUNDITY & SURVIVAL

###############################################

adjust_offspring_count <- function(a1p1,a2p1,a1p2,a2p2, base_offspring) {

if ((a1p1=="B" && a2p1=="B") && (a1p2=="B" && a2p2=="B")) return(ceiling(base_offspring * 0.3))

if ((a1p1=="B" && a2p1=="B") || (a1p2=="B" && a2p2=="B")) return(ceiling(base_offspring * 0.8))

return(base_offspring)

}

calculate_survival <- function(allele1, allele2) {

if (allele1=="B" && allele2=="B") return(0.5)

return(1)

}

###############################################

# OFFSPRING PRODUCTION

###############################################

produce_offspring <- function(parent1, parent2, base_offspring) {

num_offspring <- adjust_offspring_count(

parent1$allele1, parent1$allele2,

parent2$allele1, parent2$allele2,

base_offspring

)

offspring <- data.frame(

allele1 = character(num_offspring),

allele2 = character(num_offspring),

stringsAsFactors = FALSE

)

for (i in 1:num_offspring) {

offspring$allele1[i] <- sample(c(parent1$allele1, parent1$allele2),1)

offspring$allele2[i] <- sample(c(parent2$allele1, parent2$allele2),1)

}

return(offspring)

}

###############################################

# INITIAL POPULATIONS

###############################################

lab_population <- data.frame(

ID = paste0("111", 1:2),

parent1 = c(NA, NA),

parent2 = c(NA, NA),

allele1 = c(lab_alleles_pair1[1], lab_alleles_pair2[1]),

allele2 = c(lab_alleles_pair1[2], lab_alleles_pair2[2]),

generation_lived = 1

)

initial_wild_population_size <- 100

wild_population <- data.frame(

ID = paste0("222", 1:initial_wild_population_size),

parent1 = NA,

parent2 = NA,

allele1 = sample(initial_alleles_wild, initial_wild_population_size, replace=TRUE),

allele2 = sample(initial_alleles_wild, initial_wild_population_size, replace=TRUE),

generation_lived = 1

)

lab_data <- list()

wild_data <- list()

lab_allele_frequencies <- list()

wild_allele_frequencies <- list()

###############################################

# SIMULATION LOOP

###############################################

for (gen in 1:generations) {

cat("\nGeneration", gen, "\n")

############################

# LAB

############################

new_lab_offspring <- data.frame()

if (nrow(lab_population) > 1) {

# Sexual selection FIRST

lab_mating_pool <- apply_sexual_selection(lab_population)

# Must have 2+ individuals

if (nrow(lab_mating_pool) > 1) {

# Random pairing

idx <- sample(1:nrow(lab_mating_pool))

if (length(idx) %% 2 != 0) idx <- idx[-1]

pairs <- matrix(idx, ncol=2, byrow=TRUE)

for (k in 1:nrow(pairs)) {

p1 <- lab_mating_pool[pairs[k,1],]

p2 <- lab_mating_pool[pairs[k,2],]

# Inbreeding avoidance

r <- calculate_relatedness(p1, p2)

if (runif(1) > inbreeding_mating_probability(r)) next

# Produce offspring

offspring <- produce_offspring(p1, p2, offspring_per_pair_lab)

# Assign IDs

start_id <- max(as.numeric(sub("111","", lab_population$ID)), na.rm=TRUE) + 1

end_id <- start_id + nrow(offspring) - 1

offspring <- cbind(

ID = paste0("111", start_id:end_id),

parent1 = p1$ID,

parent2 = p2$ID,

offspring,

generation_lived = 1

)

new_lab_offspring <- rbind(new_lab_offspring, offspring)

}

}

}

# Survival + ageing

survival_probs <- mapply(calculate_survival, lab_population$allele1, lab_population$allele2)

lab_population <- lab_population[runif(nrow(lab_population)) < survival_probs, ]

lab_population$generation_lived <- lab_population$generation_lived + 1

lab_population <- lab_population[lab_population$generation_lived <= 3, ]

# Add offspring

lab_population <- rbind(lab_population, new_lab_offspring)

if (nrow(lab_population) > lab_capacity) {

lab_population <- lab_population[sample(1:nrow(lab_population), lab_capacity), ]

}

# Release 20% to wild

num_release <- round(nrow(lab_population) * 0.2)

release_inds <- lab_population[sample(1:nrow(lab_population), num_release), ]

wild_population <- rbind(wild_population, release_inds)

############################

# WILD

############################

new_wild_offspring <- data.frame()

if (nrow(wild_population) > 1) {

# Sexual selection first

wild_mating_pool <- apply_sexual_selection(wild_population)

if (nrow(wild_mating_pool) > 1) {

idx <- sample(1:nrow(wild_mating_pool))

if (length(idx) %% 2 != 0) idx <- idx[-1]

pairs <- matrix(idx, ncol=2, byrow=TRUE)

for (k in 1:nrow(pairs)) {

p1 <- wild_mating_pool[pairs[k,1],]

p2 <- wild_mating_pool[pairs[k,2],]

# Inbreeding avoidance

r <- calculate_relatedness(p1, p2)

if (runif(1) > inbreeding_mating_probability(r)) next

offspring <- produce_offspring(p1, p2, offspring_per_pair_wild)

start_id <- max(as.numeric(sub("222","", wild_population$ID)), na.rm=TRUE) + 1

end_id <- start_id + nrow(offspring) - 1

offspring <- cbind(

ID = paste0("222", start_id:end_id),

parent1 = p1$ID,

parent2 = p2$ID,

offspring,

generation_lived = 1

)

new_wild_offspring <- rbind(new_wild_offspring, offspring)

}

}

}

# Survival & age

survival_probs <- mapply(calculate_survival, wild_population$allele1, wild_population$allele2)

wild_population <- wild_population[runif(nrow(wild_population)) < survival_probs, ]

wild_population$generation_lived <- wild_population$generation_lived + 1

wild_population <- wild_population[wild_population$generation_lived <= 3, ]

wild_population <- rbind(wild_population, new_wild_offspring)

if (nrow(wild_population) > wild_capacity) {

wild_population <- wild_population[sample(1:nrow(wild_population), wild_capacity), ]

}

###############################################

# TRACK FREQUENCIES & SAVE DATA

###############################################

lab_allele_frequencies[[gen]] <- table(c(lab_population$allele1, lab_population$allele2)) /

(2 * nrow(lab_population))

wild_allele_frequencies[[gen]] <- table(c(wild_population$allele1, wild_population$allele2)) /

(2 * nrow(wild_population))

lab_data[[gen]] <- lab_population

wild_data[[gen]] <- wild_population

}

###############################################

# EXPORT TO EXCEL

###############################################

library(openxlsx)

write.xlsx(lab_data, "lab.xlsx")

write.xlsx(wild_data, "wild.xlsx")

write.xlsx(lab_allele_frequencies, "lab_allele_frequencies.xlsx")

write.xlsx(wild_allele_frequencies, "wild_allele_frequencies.xlsx")

##########

# Load required packages

library(readxl)

library(dplyr)

# Define the path to your Excel file

file_path <- "wild_allele_frequencies.xlsx"

# Get the sheet names from the Excel file

sheet_names <- excel_sheets(file_path)

# Initialize an empty data frame to store the combined data

combined_data <- data.frame()

# Loop through each sheet and combine the data

for (sheet in sheet_names) {

# Read the data from the current sheet

sheet_data <- read_excel(file_path, sheet = sheet)

# Add a new column to the data indicating the sheet name

sheet_data$Sheet_Name <- sheet

# Bind the current sheet's data to the combined data

combined_data <- bind_rows(combined_data, sheet_data)

}

# View the combined data

head(combined_data)

# Optionally, save the combined data to a new Excel file

write.xlsx(combined_data, "combined_data_wild.xlsx")

# Define the path to your Excel file

file_path1 <- "lab_allele_frequencies.xlsx"

# Get the sheet names from the Excel file

sheet_names <- excel_sheets(file_path1)

# Initialize an empty data frame to store the combined data

combined_data <- data.frame()

# Loop through each sheet and combine the data

for (sheet in sheet_names) {

# Read the data from the current sheet

sheet_data <- read_excel(file_path1, sheet = sheet)

# Add a new column to the data indicating the sheet name

sheet_data$Sheet_Name <- sheet

# Bind the current sheet's data to the combined data

combined_data <- bind_rows(combined_data, sheet_data)

}

# View the combined data

head(combined_data)

# Optionally, save the combined data to a new Excel file

write.xlsx(combined_data, "combined_data_lab.xlsx")
